# Integrated ceRNA Network Analysis in Silica-Induced Pulmonary Fibrosis and Discovery of miRNA Biomarkers

**DOI:** 10.3390/toxics14010063

**Published:** 2026-01-09

**Authors:** Jia Wang, Yuting Jin, Qianwei Chen, Fenglin Zhu, Min Mu

**Affiliations:** 1 Key Laboratory of Industrial Dust Prevention and Control, Occupational Safety and Health, Ministry of Education, Anhui University of Science and Technology, Huainan 232000, China; wangjia@aust.edu.cn (J.W.); 2020081@aust.edu.cn (Q.C.); 2 School of Public Health, Anhui University of Science and Technology, Hefei 231131, China; emily002021@163.com; 3 Joint National-Local Engineering Research Centre for Safe and Precise Coal Mining, Anhui University of Science and Technology, Huainan 232000, China

**Keywords:** silicosis, ceRNA network, miRNA biomarkers, transcriptomic sequencing

## Abstract

Silicosis is an irreversible and progressive pulmonary fibrotic disease caused by the long-term inhalation of silica dust. The precise molecular mechanisms underlying the disease remain incompletely understood, and effective early diagnostic biomarkers are still lacking. In this study, we used a silicosis mouse model and transcriptomic sequencing to identify 2950 mRNAs, 461 lncRNAs, 81 miRNAs, and 44 circRNAs that were differentially expressed in lung tissue. Enrichment analysis revealed that these differentially expressed genes were significantly enriched in the phosphatidylinositol 3-kinase (PI3K)–protein kinase B (Akt) signaling pathway, nuclear factor kappa-light-chain-enhancer of activated B cell (NF-κB) signaling pathway, and tumor necrosis factor (TNF) signaling pathway. The constructed competing endogenous RNA (ceRNA) network highlighted extensive regulatory interactions among lncRNAs/circRNAs, miRNAs, and mRNAs. Human validation showed that the expression levels of hsa-miR-215-5p and hsa-miR-146b-5p were significantly upregulated in the peripheral blood of early-stage pneumoconiosis patients, while hsa-miR-485-5p was downregulated. Logistic regression analysis revealed that hsa-miR-215-5p (OR = 1.966, 95% CI: 1.6938–2.2796, *p* < 0.001) and hsa-miR-146b-5p (OR = 1.9367, 95% CI: 1.697–2.201, *p* < 0.001) were independent risk factors for pneumoconiosis (*p* < 0.001). ROC curve analysis showed that both miRNAs demonstrated good diagnostic efficacy for pneumoconiosis, with AUC values of 0.9563 and 0.8876, respectively. These results provide novel insights into the complex ceRNA regulatory network involved in silicosis pathogenesis and suggest potential early, non-invasive diagnostic biomarkers.

## 1. Introduction

Silicosis is a systemic disease characterized by nodular fibrosis of lung tissue, primarily caused by the prolonged inhalation of free silica (SiO_2_) dust particles during occupational activities [1,2]. As a type of pneumoconiosis, it is one of the most common and severe forms of occupational disease. Silicosis is notable for its latent, delayed, and progressive nature, coupled with its high mortality rates [3]. Once contracted, the disease continues to progress even if the individual is removed from the dusty environment, and there are currently no effective treatments available [4]. This situation poses significant economic and health burdens on patients. Significant efforts over recent decades to prevent and control silicosis have led to some positive outcomes [5]. For instance, while the global burden of silicosis showed a declining trend from 1990 to 2019, the absolute number of new cases has increased [4,6,7]. This increase is especially pronounced in developing countries, where the incidence continues to rise. In countries such as Brazil, India, and China, tens of millions of workers are exposed to dust daily in high-risk industries like mining, quarrying, construction, and others [6,7,8,9]. At the same time, developed countries are not immune to the significant risks of silicosis [7]. For example, the United States reported 2512 silicosis-related deaths between 1999 and 2018 [10]. Consequently, silicosis remains a pressing concern for occupational health and safety worldwide.

Although the pathogenesis of silicosis has been studied extensively, the exact mechanisms remain incompletely understood, and effective clinical treatments are lacking [5,11]. Current research indicates that the primary pathogenesis of silicosis is closely linked to macrophage damage caused by the inhalation of silica dust [12]. Once silica dust accumulates in the lungs, it results in macrophage destruction and the release of various chemokines, cytokines, and inflammatory mediators [3]. These inflammatory responses trigger further inflammation in lung tissue, promote fibroblast proliferation, and lead to the deposition of extracellular matrix, ultimately resulting in pulmonary fibrosis [13,14,15]. Subacute exposure to silica nanoparticles can trigger autophagic dysfunction and pulmonary inflammation, thereby inducing pulmonary epithelial–mesenchymal transition via the activation of the p62/NF-κB signaling pathway [16]. Regulation of the PI3K/Akt/mechanistic target of rapamycin (mTOR) pathway by silica dust exposure influences macrophage autophagy induction and inflammatory cytokine concentrations, thereby affecting the transition of human fetal lung-1 (HFL-1) cells toward a myofibroblast phenotype induced by silica dust [17]. In a murine model of silicosis, the macrophage necroptosis pathway is markedly activated and promotes disease progression by augmenting pulmonary inflammation and fibrotic responses, a process reversible by the receptor-interacting protein 1 kinase inhibitor necrostatin-1 [18]. Additionally, epigenetics plays critical regulatory roles in the onset and progression of silicosis, including DNA methylation, non-coding RNAs (ncRNAs), and histone modifications [19,20]. For instance, the analysis of DNA methylation profiles in silicosis lung tissue revealed that, in advanced silicosis, CpG methylation sites in the promoters of phosphatase and tensin homolog were deleted on chromosome ten (PTEN) and c-Jun increased, and the hypermethylation of the PTEN promoter was correlated with reduced PTEN protein expression [21]. In a silicosis model, exposure to silica induces significant alterations in histone H3K4 and H3K27 methylation levels during lung fibroblast transdifferentiation, primarily characterized by H3K27 demethylation and H3K4 methylation [22].

Whole-transcriptome technology allows for a comprehensive bioinformatics analysis of both coding and non-coding RNAs, facilitating the investigation of gene expression changes and the regulatory roles of non-coding RNAs [23,24]. This approach allows us to explore and interpret the transcriptional and regulatory mechanisms underlying biological phenomena. In particular, competitive endogenous RNA (ceRNA) regulatory networks have been employed to elucidate the molecular mechanisms influenced by diseases and environmental pollutants [25,26]. Recent studies have indicated that certain miRNAs, lncRNAs, and circRNAs are closely associated with the development of pneumoconiosis, showing potential as non-invasive biomarkers and prognostic indicators [24,27]. At the transcriptional level, non-coding RNAs can recruit chromatin-modifying complexes to specific genomic regions, thereby altering histone modifications and DNA methylation patterns, which subsequently affect chromatin accessibility and gene expression [24]. For instance, the long non-coding RNA LINC03047, through RELA-mediated transcriptional activation, regulates SLC39A14 expression, driving silica-induced ferroptosis and epithelial–mesenchymal transition [28]. Additionally, high expression of circPVT1 promotes epithelial–mesenchymal transition (EMT) and enhances the migratory ability of silica-treated epithelial cells [29]. At the post-transcriptional level, non-coding RNAs finely regulate gene expression by modulating mRNA stability, mediating alternative splicing, and influencing translation. For example, elevated expression of miR-7219-3p promotes fibroblast-to-myofibroblast transition (FMT), cell migration, and proliferation by targeting SPRY1 and activating the RAS/ERK/MAPK pathway [30]. Meanwhile, miR-411-3p effectively reduced pulmonary silicosis in mouse lungs by regulating myocardia-related transcription factor (Mrtf) expression [31], whereas miRNA-34c-5p inhibits silica-induced pulmonary fibrosis by targeting Fra-1 via the p53 and PTEN/PI3K/Akt signaling pathways [32]. Regulation through ceRNA networks is more complex, centered on the competition among different RNA molecules that share miRNA response elements for binding to the same miRNA, thereby mutually influencing each other’s stability or translational efficiency [24]. For instance, lncRNA UCA1 modulates the EMT process by competitively binding to miR-204-5p, leading to the release of its target gene Zinc Finger E-Box Binding Homeobox 1 (ZEB1) [33]. Similarly, circHIPK3 functions as a sponge for miR-338-3p, enhancing the expression of SRY-related HMG-Box gene 4 (SOX4) and collagen type I alpha 1 chain (COL1A1), thereby promoting fibroblast-to-myofibroblast differentiation [34]. However, the regulatory mechanisms of non-coding RNAs are highly complex, particularly with respect to the interactions between mRNAs, miRNAs, lncRNAs, and circRNAs, which are still not fully understood. The regulatory networks involving these non-coding RNAs, including the interactions between miRNAs, lncRNAs, and circRNAs, need further investigation [35].

In this study, we used a previously established silica-induced silicosis mouse model [36]. First, we performed lung function tests to evaluate changes in lung performance in the model mice and conducted hematoxylin and eosin (H&E) staining to confirm the model’s reliability. Next, we analyzed lung tissue through full-transcriptome sequencing, providing a comprehensive examination of the regulatory networks involving silicosis-related genes, including miRNAs, mRNAs, lncRNAs, and circRNAs. This analysis included differential expression profiling and the construction of a ceRNA network to identify key interactions among these molecules. Furthermore, the expression levels of candidate miRNAs in human peripheral blood and their effectiveness as diagnostic biomarkers for pneumoconiosis were examined using a case–control study design. These results provide novel insights into the complex ceRNA regulatory network involved in silicosis pathogenesis and the identification of early non-invasive diagnostic biomarkers.

## 2. Materials and Methods

### 2.1. Animals and Silica

Male C57BL/6 J mice (6–8 weeks old) were purchased from the Chang Zhou Cavens Laboratory Animal Ltd. (Changzhou, China) (license number: SCXK (Su) 2021-0013). They were housed in a pathogen-free environment (light–dark cycle: 12/12 h; temperature: 22–24 °C; relative humidity: 45–65%). Food and water were provided ad libitum. Crystalline silica particulates were purchased from Sigma Aldrich (St. Louis, MO, USA) (Cat#S5631), with 80% of the particles having a diameter between 1 and 5 µm. In addition, silica particulates were suspended in sterile phosphate-buffer saline, autoclaved for 30 min, and sonicated for 10 min before use. All animal studies were conducted in accordance with the National Institutes of Health’s Guide for the Care and Use of Laboratory Animals (NIH Publication No. 8023, revised 1978) and were licensed by the Institutional Review Board (IRB) for Biomedical Research of Anhui University of Science and Technology (No: S2-2023-018).

### 2.2. Establishment of a Silicosis Mouse Model

The silicosis mouse model was established through the repeated inhalation of silica dust via the nose, as previously described [36]. Briefly, 20 mice were randomly divided into two groups: the vehicle control group (CK) and the silica-treated group (SiO_2_). The mice received either a silica suspension (20 µg/µL, 80 µL) or phosphate-buffered saline (PBS) (80 µL) by nasal drops once a day for 16 days. On day 31, the pulmonary function of the mice was tested before they were euthanized.

The procedure was conducted as follows: Mice were anesthetized using isoflurane, and their respiratory status was continuously monitored throughout the anesthesia. Once their breathing changed from shallow and rapid to deep and slow (approximately 2–3 times slower than normal), the instillation procedure was initiated immediately. During the operation, the mouse was positioned laterally across the experimenter’s palm, supporting its body with the third metacarpophalangeal joint. The thumb was gently placed against the mandible to maintain head elevation while avoiding pressure on the throat, which could trigger a swallowing reflex. Instillation was carefully synchronized with the mouse’s respiratory rhythm: a silica suspension (20 µg/µL, 80 µL) or an equal volume of PBS was administered dropwise along one side of the nasal ala at the precise transition from exhalation to inhalation, continuing in aliquots until the target volume was delivered. Following the instillation, pronounced moist rales could be auscultated over the lung fields. Subsequently, gentle thoracic massage was performed to promote the dispersion and redistribution of the liquid within the lungs. After the procedure, each mouse was individually housed and continuously monitored until it fully recovered from anesthesia and restored voluntary activity.

### 2.3. Pulmonary Function Test for Mice

Mouse lung function was measured using whole-body plethysmograph (WBP-4MR, Shanghai TOW Intelligent Tech. Co., Ltd., Shanghai, China). The specific steps were followed according to the instrument’s instructions. Within 4 days from detection, the mice were placed in the whole-body plethysmograph for 45 min each day. On the test day, after the mice acclimated to the chamber for 65 min, respiratory parameters were tested, including inspiratory time (Ti), tidal volume (Tv), expiratory time (Te), and respiratory rate.

### 2.4. Lung Tissue Collection and Treatment in Mice

Following established methods, mice were euthanized, and lung tissue was obtained [36]. The lung tissue was randomly divided into two parts. One part was fixed by soaking in 4% paraformaldehyde for more than 24 h. After fixation, paraffin embedding and sectioning were performed. The other part of lung tissue was quickly frozen and stored in liquid nitrogen at −80 °C for RNA sequencing and RT-qPCR.

### 2.5. Section Staining

Four µm-thick paraffin-embedded lung tissue was sectioned by deparaffinizing, rehydrating, and staining with H&E. The staining process mainly included the following steps: removing paraffin from the slices using xylene, treating them with alcohol distilled water, staining them with hematoxylin, treating them with distilled water, staining them with eosin, dehydrating them till they are transparent, and sealing them for preservation.

### 2.6. Transcriptome Sequencing and Analysis

Total RNA was extracted from the lung tissue using TRIzol^®^ reagent according the manufacturer’s instructions (Invitrogen, Carlsbad, CA, USA). longRNA-seq library and smallRNA-seq library were prepared using TruSeqTM Stranded Total RNA Library (or Small RNA sample) Prep Kit from Illumina (San Diego, CA, USA). After quantification, the RNA-seq sequencing library was sequenced with the Illumina NovaSeq X Plus at Shanghai Majorbio Bio-pharm Biotechnology Co., Ltd. (Shanghai, China). Quality control of the raw paired-end reads was performed using Fastp (Version 0.19.5) and fastx_toolkit (Version 0.0.14). The clean reads were aligned to the reference genome using HISAT2 (Version 2.1.0). The mapped reads were assembled by StringTie (Version 1.3.3b). Genes and transcripts were compared and annotated in Non-Redundant Protein Database (NR), Swiss-Prot, Protein Family (Pfam), Evolutionary Genealogy of Genes: Non-supervised Orthologous Groups (EggNOG), Gene Ontology (GO), and Kyoto Encyclopedia of Genes and Genomes (KEGG) databases. The NONCODE (http://www.noncode.org/index.php, accessed on 1 December 2023) and miRBase22.0 databases were used to identify the mappings for miRNAs and lncRNAs, respectively. The CIRI2 (CircRNA Identifier) and find_circtools were used to identify circRNAs. The expression level of each gene, miRNA, and lncRNA was calculated according to the transcripts per million (TPM) method, while the expression level of each circRNA was calculated using the reads per million mapped reads (RPM) method. For circRNA quantification, the analytical focus is not on the full transcript but on the back-splice junction (BSJ) site, where each sequencing read spanning the BSJ corresponds to one circRNA molecule. Typically, only BSJ coordinates are reliably known, while the precise full-length sequence and transcript length of circRNAs (particularly intron-derived circRNAs) remain challenging to define accurately. Since the TPM calculation depends on the exact transcript length, RPM is regarded as a more appropriate metric for circRNA quantification in this context. RSEM was used to quantify ncRNA and gene abundances. Differential expression analysis was performed using the DESeq2 with |log2 (foldchange)| ≥ 1 and *p*-adjust ≤ 0.05.

Additionally, target gene and ncRNA predictions of miRNAs were performed using miRanda (http://www.miranda.org, accessed on 16 December 2023). To understand the biological functions of the target genes, GO functional enrichment analysis, KEGG pathway analysis, and Gene Set Enrichment Analysis (GSEA) were carried out by the Goatools (Version 0.6.5), KOBAS (Version 2.1.1), and GSEA (Version 3.0) software. Based on the expression levels of mRNA and ncRNA, the Spearman correlation coefficient was used to evaluate the correlation between DE mRNAs and DE ncRNAs. The ceRNA network (lncRNA-miRNA-mRNA and circRNA-miRNA-mRNA) was built and visually displayed by the Cytoscape software (Version 3.10.2).

### 2.7. Study Participants

Study participants were recruited from a central hospital in Northern China. The inclusion criteria were as follows: (1) for the case group: patients with a confirmed imaging diagnosis of stage I pneumoconiosis, based on the Chinese Criteria for the Diagnosis of Occupational Injuries and Diseases [37]; for the control group: individuals without pneumoconiosis; (2) voluntary participation in the survey with complete relevant data; (3) age between 45 and 55 years; and (4) completion of pulmonary function tests and physical examination. The exclusion criteria were as follows: (1) a prior diagnosis of lung cancer, pulmonary tuberculosis, or other pulmonary diseases, and (2) the unavailability of peripheral blood biospecimens.

A total of 135 participants were included in the final analytical cohort, comprising 62 pneumoconiosis cases and 73 controls. Detailed information is presented in Table S1. All participants provided written informed consent prior to enrollment. The study protocol was reviewed and approved by the Institutional Review Board (IRB) for Biomedical Research of Anhui University of Science and Technology (Approval No: S2-2023-018).

### 2.8. Pulmonary Function Testing in Human Subjects

Pulmonary function testing was performed by trained staff using a Japanese chest spirometer. The measured parameters included forced vital capacity (FVC), forced expiratory volume in one second (FEV1), percentage of predicted forced vital capacity (FVC%), percentage of predicted forced expiratory volume in one second (FEV1%), and the FEV1/FVC ratio. The spirometer was calibrated daily prior to measurements. Participants were required to perform the test three times in accordance with the standards recommended by the American Thoracic Society, with a 10 min interval between each test. The best result from the three trials was recorded for analysis. All respiratory function tests were conducted between 8:00 AM and 12:00 PM to minimize the influence of diurnal variation.

### 2.9. Collection and Processing of Human Peripheral Blood Samples

During the physical examination, 5 mL of whole blood was collected using EDTA-anticoagulated tubes. The tubes were gently inverted to mix the contents. Subsequently, 250 µL of the whole blood was aliquoted into a 1.5 mL RNase-free microcentrifuge tube. Then, 750 µL of TRIzol reagent was added to the tube, followed by rapid and gentle inversion for thorough mixing. The prepared samples were immediately stored at −80 °C for future use.

### 2.10. Quantitative Real-Time PCR (RT-qPCR) Validation

The expression levels of DEmRNAs, DElncRNAs, and DEmiRNAs were validated by RT-qPCR. Total RNA was extracted from lung tissue using TRIzol^®^ reagent according to the manufacturer’s instructions (Invitrogen, Carlsbad, CA, USA). RNA concentration and purity were subsequently measured using a Nanodrop 2000 spectrophotometer. Reverse transcription of mRNA, lncRNA, circRNA, and miRNA was performed using the First-Strand cDNA Synthesis Kit (Beijing, China) (Cat. Nos. KR211, KR202, and KR136, respectively). Amplification and detection were carried out on an Applied Biosystems QuantStudio 3 Real-Time PCR System using the Tiangen SuperReal PreMix Plus (SYBR Green) Kit (Beijing, China) (Cat. Nos. FP207, FP411, and FP402).

Primers were designed using the Primer 5 and miRprimer2 software. For miRNA quantification, forward primers were designed using the polyadenylation-coupled reverse-transcription method, with universal reverse primers supplied in the commercial kit. To ensure the specific amplification of circRNAs, all corresponding primers were designed to span the back-splice junction. The precise coordinates of each back-splice site were determined, and 200–300 bp of genomic sequence flanking the junction (both upstream and downstream) was extracted. Primers were positioned within these flanking regions so that the resulting amplicon necessarily encompassed the back-splice site, thereby unambiguously distinguishing circRNAs from their linear transcript counterparts. To further improve the specificity of circRNA detection, total RNA was pretreated with RNase R (5 µg RNA incubated with 20 U/µL RNase R at 37 °C for 30 min to degrade linear RNA) before reverse transcription and subsequent steps. After design, all primer pairs were screened for genome-wide specificity using NCBI Primer-BLAST (https://www.ncbi.nlm.nih.gov/tools/primer-blast/, accessed on 12 March 2024) to eliminate potential non-specific binding. Each pair was then validated in preliminary experiments; only those showing normal amplification kinetics and a single peak in melt-curve analysis were selected for final use. The Hprt gene was selected as the reference for normalizing DEmRNAs, DElncRNAs, and DEcircRNAs, while U6 was used as the reference for normalizing DEmiRNAs. Relative expression of target genes was calculated using the 2^−ΔΔCt^ method. All RT-qPCR assays were performed with three biological replicates and three technical replicates. Primers used are listed in Table S2.

### 2.11. Data Analysis

Statistical analysis was performed using the IBM SPSS Statistics 27.0 (Armonk, NY, USA) and GraphPad Prism software 9.4.1 (Boston, MA, USA). Continuous variables conforming to a normal distribution were presented as the mean ± standard deviation ($\bar{x}$ ± s), while categorical variables were expressed as counts (percentages), *n* (%). Differences between groups were analyzed using the two-sample t-test or one-way analysis of variance (ANOVA) for normally distributed data. For non-normally distributed data, results were expressed as the median and were analyzed using the Mann–Whitney U test. The relationship between miRNA relative expression levels and the risk of pneumoconiosis was assessed via binary logistic regression analysis. The dependent variable was the presence or absence of pneumoconiosis. Independent variables included age, body mass index (BMI), educational level (below high school, college or above), annual household income (<CNY 200,000, ≥CNY 200,000), smoking status (yes/no), drinking status (yes/no), shift work status (yes/no), hypertension (yes/no), and hyperlipidemia (yes/no). The diagnostic efficacy of miRNAs for pneumoconiosis was evaluated using receiver operating characteristic (ROC) curve analysis, and the area under the curve (AUC) was calculated. A *p*-value < 0.05 was considered statistically significant.

## 3. Results

### 3.1. Silica Exposure Caused Pulmonary Fibrosis and Decreased Respiratory Function in Mice

**Figure 1:**
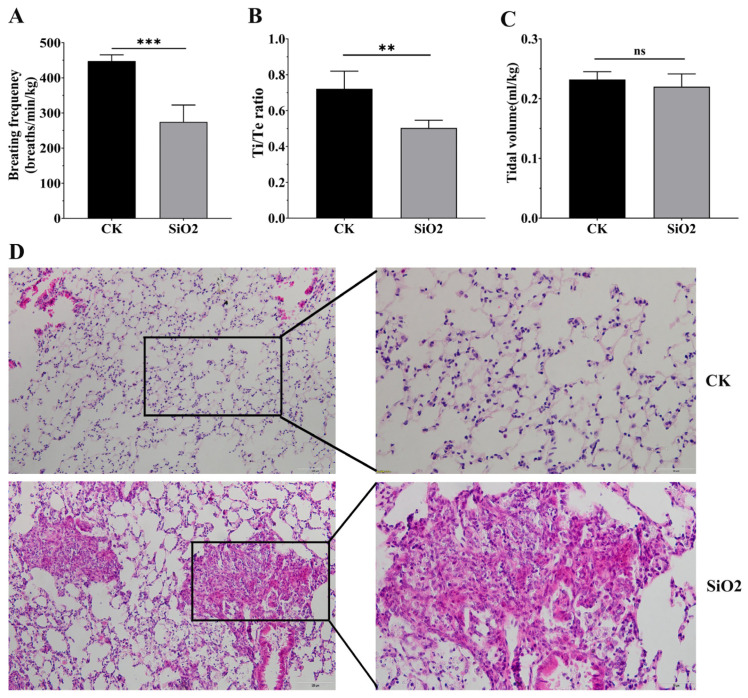
Silica exposure induces pulmonary fibrosis and impairs respiratory function in mice. (**A**) Pulmonary tidal volume after exposure of mice to SiO_2_ particles. (**B**) Breathing frequency after exposure of mice to SiO_2_ particles. (**C**) Ti/Te ratio after exposure of mice to SiO_2_ particles (*n* = 6, ** *p* < 0.01, *** *p* < 0.001 vs. the saline group). (**D**) Representative H&E staining of lung tissue from different groups (400× magnification, scale bar = 50 µm; 200× magnification, scale bar = 100 µm) (CK, saline group; SiO_2_, on day 31 of silica dust exposure).

To analyze the effects of silica dust exposure on lung function, whole-body plethysmograph was used to detect respiratory parameters of mice. The results showed that the respiratory rate and Ti/Te (inspiratory time/expiratory time) ratio in the SiO_2_-treated group were significantly lower than those in the control group (Figure 1A,B). While tidal volume did not change significantly, it remained smaller than that in the control group, indicating compromised lung function in the SiO_2_-treated mice (Figure 1C).

H&E staining revealed well-defined lung tissue with intact alveolar walls in the control group (Figure 1D). Conversely, lung tissue from SiO_2_-treated mice displayed significant damage, including disrupted alveolar walls, inflammatory cell infiltration, and fibrotic nodules in some areas. These findings demonstrated that silica exposure caused significant damage to lung tissue in mice, with fibrotic structures appearing at post-exposure day 31. This damage prevents the lung tissue from maintaining its normal physiological function, leading to reduced lung capacity.

### 3.2. Characterization of Sequencing Data in Lungs

**Figure 2:**
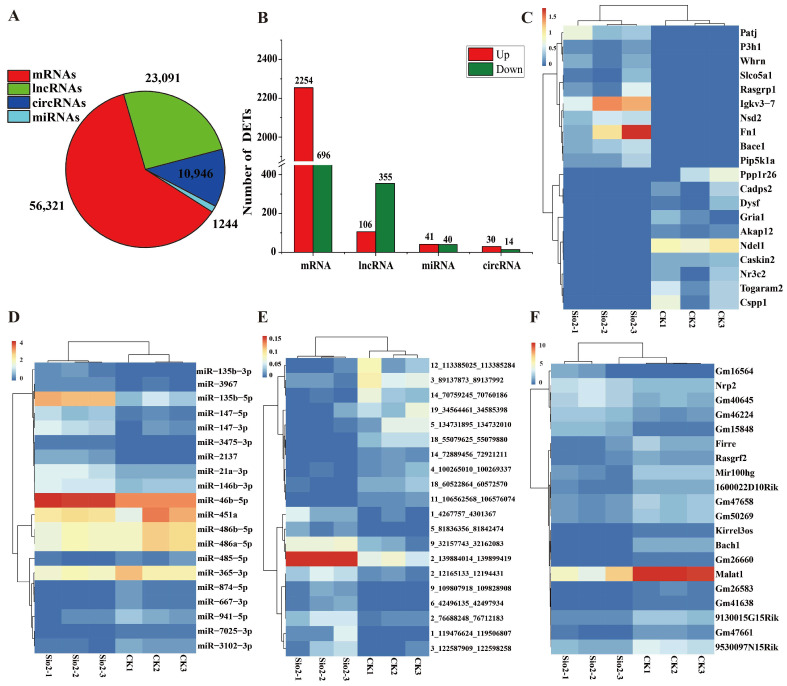
Characterization of mRNA, lncRNA, miRNA, and circRNA in mouse lung tissue under silica exposure. (**A**) Distribution of mRNA, lncRNA, miRNA, and circRNA counts. (**B**) Distribution of numbers of differentially expressed mRNA, lncRNA, miRNA, and circRNA. (**C**) Top 20 differentially expressed mRNA. (**D**) Top 20 differentially expressed miRNA. (**E**) Top 20 differentially expressed circRNA. (**F**) Top 20 differentially expressed lncRNA.

For longRNA sequencing (mRNA, lncRNA, circRNA), a total of 120.73 GB of clean data was obtained from six samples, with a Q30 percentage exceeding 95.42% (Table S3). The clean reads of each sample were compared to the reference genome sequence, yielding a mapping rate ranging from 93.8% to 96.54%. Additionally, for smallRNA sequencing, six samples obtained 64.67 MB raw reads, with each sample providing over 10.19 MB and a Q30 percentage higher than 96.04%. The mapping efficiency ranged from 96.42% to 97.90% (Table S4). All sequencing data are available for subsequent analysis. Through the high-quality data assembly, identification, and prediction, in total 56,231 mRNAs, 23,091 lncRNAs, 10,946 circRNAs, and 1244 miRNAs were identified (Figure 2A).

### 3.3. Analysis of Differential Expression Genes

With differential expression analysis by DESeq2, in total 2950 mRNAs (2254 upregulated, 696 downregulated), 461 lncRNAs (106 upregulated, 355 downregulated), 81 miRNA (41 upregulated, 40 downregulated), and 44 circRNA (30 upregulated, 14 downregulated) were obtained as significant differentially expressed between the vehicle control group (CK) and silica-treated group (SiO_2_), respectively (Figure 2B, Tables S5–S8). Notably, the number of upregulated genes was greater than the number of downregulated genes, indicating that mice adapted to silica exposure primarily through positive regulation. Meanwhile, the top 20 mRNA, miRNA, lncRNA and circRNA with different expression levels were analyzed (Figure 2C–F).

### 3.4. Enrichment Analysis of DEmRNAs, DEcircRNA, DElncRNAs, and DEmRNAs

**Figure 3:**
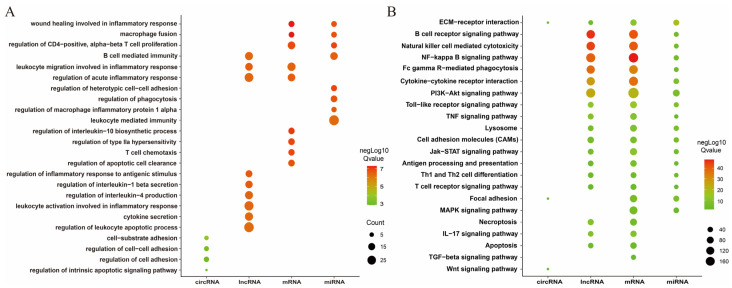
Enrichment analysis of differentially expressed mRNAs (DEmRNAs), target genes of DEmiRNAs, target genes of DElncRNAs, and host genes of DEcircRNAs. (**A**) GO enrichment analysis of DEmRNAs, host genes of DEcircRNAs, target genes of DElncRNAs, and DEmRNAs. (**B**) KEGG enrichment analysis of DEmRNAs, host genes of DEcircRNAs, target genes of DElncRNAs, and DEmRNAs.

To investigate the biological functions of differentially expressed genes (DEmRNAs), DEmiRNA target genes, DElncRNA target genes, and DEcircRNA chost genes, GO and KEGG enrichment analyses were performed. The GO enrichment analysis showed that these genes were enriched in different GO terms (Figure 3A). For example, the circRNA host genes were mainly enriched in cell–substrate adhesion, regulation of cell–cell or cell adhesion, and regulation of intrinsic apoptotic signaling pathway. The miRNA target genes were mainly enriched in B cell-mediated immunity, leukocyte-mediated immunity, macrophage fusion, and regulation of CD4-positive and alpha-beta T cell proliferation. The lncRNA target genes were enriched in B cell-mediated immunity, cytokine secretion, leukocyte activation involved in inflammatory response, and regulation of leukocyte apoptotic process. The DEGs were mainly enriched in macrophage fusion, regulation of acute inflammatory response, and regulation of apoptotic cell clearance. KEGG enrichment analysis indicated that these genes were mainly involved in extracellular matrix (ECM)–receptor interaction, cytokine–cytokine receptor interaction, B cell receptor signaling pathway, PI3K-Akt signaling pathway, TNF signaling pathway, Transforming Growth Factor (TGF)-beta signaling pathway, necroptosis, apoptosis, and Th1 and Th2 cell differentiation (Figure 3B). At the same time, we also noted that circRNA host genes are mainly involved in three metabolic pathways, while the enrichment results of other gene sets were less different.

In addition, we performed GSEA enrichment analysis for all genes. The results showed that the most enriched genes sets included the lysosomes, complement and coagulation cascades, antigen processing and presentation, Toll-like receptor signaling pathway, RIG-I-like receptor signaling pathway, C-type lectin receptor signaling pathway, IL-17 signaling pathway, Th17 cell differentiation, and B cell receptor signaling pathway (Figure S1).

### 3.5. Analysis of ceRNA Networks

**Figure 4:**
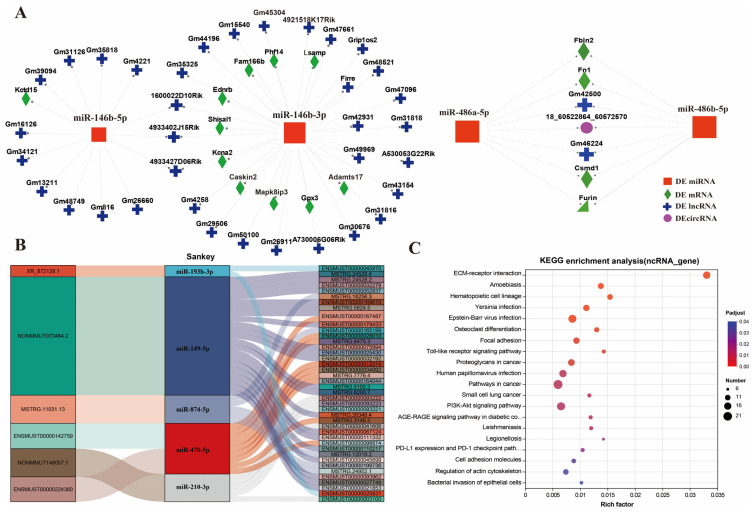
Analysis of ceRNA networks. (**A**) ceRNA networks of the miR-146b-3p/miR-146b-5p and miR-486a-5p/miR-486b-5p pairs. (**B**) Sankey diagram depicting the interactions among core lncRNAs/circRNAs, miRNAs, and mRNAs. (**C**) Functional enrichment analysis of target genes for the identified non-coding RNAs (ncRNAs).

The ceRNA networks were constructed from differentially expressed miRNAs, mRNAs, lncRNAs, and circRNAs, which were composed of 40 miRNAs, 9 circRNAs, 145 lncRNAs, and 295 mRNAs. They included 10 pairs of miRNA-circRNA, 343 pairs of miRNA-mRNA, and 415 pairs of miRNA-lncRNA relationships. In addition, we obtained multiple groups of miRNA/LncRNA-mRNA-circRNA networks (Figure S2, Tables S9 and S10), for example, lncRNA Gm16126-miRNA(146b-5p)-Kctd15, circRNA(18_60522864_60572570)-miRNA(468a-5p)-Fn1, and circRNA(18_60522864_60572570)-miRNA(468b-5p)-Fbln2-lncRNAGm46224 (Figure 4A). It is evident that there are strong relationships within these ceRNAs. In particularly, some miRNAs participate in the regulation of multiple genes, while a single gene can be regulated by multiple miRNAs or LncRNA. Similar results were obtained through Sankey analysis (Figure 4B). For instance, miRNA470-5p acted on multiple LncRNAs and mRNAs simultaneously. KEGG enrichment analysis indicated that these genes of ceRNA networks were mainly involved in ECM–receptor interaction, focal adhesion, Toll-like receptor signaling pathway, PI3K-Akt signaling pathway, and NF-kappa B signaling pathway (Figure 4C).

Additionally, we constructed a PPI network for 295 key mRNA genes, which consisted of 155 nodes and 300 edges (Figure S3, Table S11). Based on the ranking of the connections between nodes in the PPI network, Gapdh ranked first, followed by Fn1, Tlr2, Cd4, Cd44, Nfkbia, Csf1r, and Hif1a. Furthermore, the expression levels of most key genes were significantly upregulated. These findings suggest that these key genes play important regulatory roles in response to silica exposure.

### 3.6. Identification of Core ncRNAs and Genes in Key Metabolic Pathways

**Figure 5:**
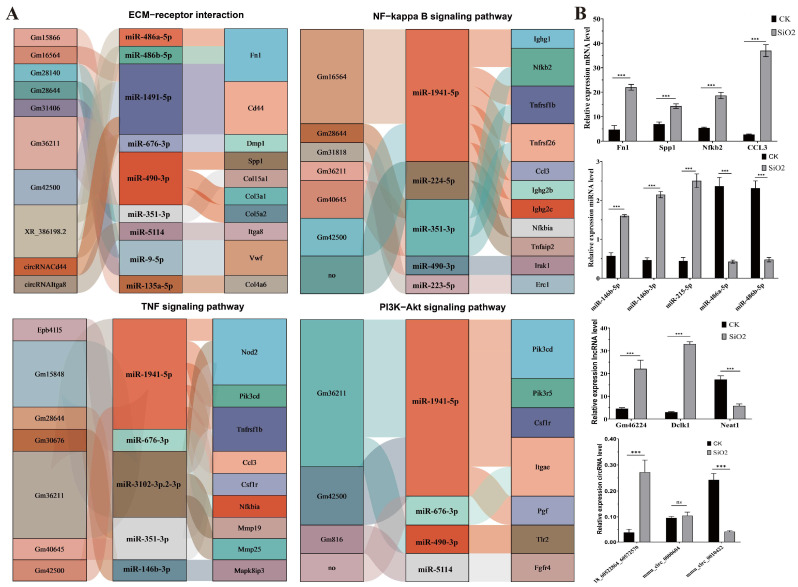
Identification of core ncRNAs and genes in key metabolic pathways. (**A**) Core ncRNAs and genes associated with the ECM–receptor interaction pathway, NF-κB signaling pathway, TNF signaling pathway, and PI3K-Akt signaling pathway. (**B**) Validation of expression levels for the core ncRNAs and genes by quantitative real-time PCR (*n* = 3, *** *p* < 0.001 vs. the saline group).

To further investigate the regulatory mechanisms of ncRNA and mRNA expression in key metabolic pathways, this study examines the relationships between differentially expressed genes and several key signaling pathways (Figure 5). The analysis reveals that nine miRNAs, eight lncRNAs, and two circRNAs regulate the expression of 10 key genes in the ECM–receptor interaction pathway. Additionally, in the NF-kappa B signaling pathway, five miRNAs and six lncRNAs modulate the expression of 11 key genes. In the TNF signaling pathway, five miRNAs and seven lncRNAs regulate the expression of nine key genes. In the PI3K-Akt signaling pathway, four miRNAs and three lncRNAs participate in regulating the expression of six key genes. Additionally, these regulatory relationships indicate that SiO_2_ exposure can affect the activity of multiple signaling pathways through the lncRNA/circRNA–miRNA–mRNA axis. For instance, miR-486a-5p, miR-490-3p, and miR-1941-5p regulate the expression of Fn1, Spp1, and CCL3, while these miRNAs are themselves modulated by the corresponding circRNAs and lncRNAs, such as cireRNACd4, Gm42500, and Gm36211 (Figure 5A).

To verify the quality and expression patterns of core ncRNAs and genes, qRT-PCR analysis was conducted on four DEmRNAs, five DEmiRNAs, three DElncRNAs, and three DEcircRNAs. The results are shown in Figure 5B. The expression levels of Fn1, Spp1, Nfkb2, CCL3, miR-146b-5p, miR-146b-3p, miR-215-5p, Gm46224, Dclk1, and 18_60522864_60572570 were significantly upregulated after SiO_2_ exposure, while miR-486a-5p, miR-486b-5p, Neat1, and mmu_circ_0010422 were significantly downregulated after SiO_2_ exposure. Only mmu_circ_0008604 changed but did not reach a significant level. In addition, these gene expression level results are similar to RNA-seq analyses, indicating that the RNA-seq data are reliable.

### 3.7. Expression Characteristics and Diagnostic Value of Candidate miRNAs in Pneumoconiosis

This study enrolled a total of 135 male participants, consisting of 62 cases (pneumoconiosis) and 73 controls. No statistically significant differences were observed between the two groups regarding age, waistline, hipline, BMI, annual household income, diabetes, smoking, or drinking consumption (*p* > 0.05), as shown in Table 1. However, significant differences were identified in educational level, prevalence of shift work, hypertension, and hyperlipidemia. Specifically, the pneumoconiosis group had a lower educational level, a higher prevalence of shift work, and significantly higher rates of hypertension and hyperlipidemia compared to the control group. Additionally, pulmonary function indicators were significantly reduced in the pneumoconiosis group. Both the forced expiratory volume in one second as a percentage of predicted value (FEV1%) and forced vital capacity as a percentage of predicted value (FVC%) were lower than those in the control group, which aligns with the expected lung function impairment caused by pneumoconiosis.

**Figure 6:**
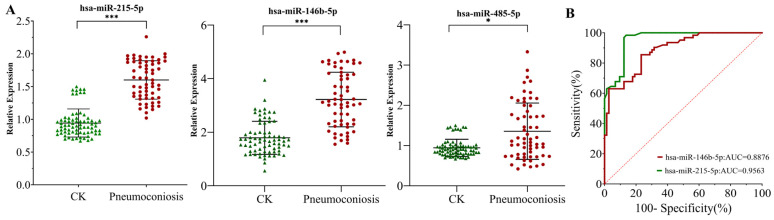
Expression profiles of candidate miRNAs in pneumoconiosis patients and controls. (**A**) Expression levels of hsa-miR-215-5p, hsa-miR-146b-5p, and hsa-miR-485-5p in peripheral blood (* *p* < 0.05, *** *p* < 0.001 vs. the control groups). (**B**) Receiver operating characteristic (ROC) curve analysis evaluating the diagnostic potential of hsa-miR-215-5p and hsa-miR-146b-5p (CK, control groups).

Through human–mouse sequence homology analysis, three differentially expressed candidate miRNAs homologous to humans were preliminarily identified, i.e., hsa-miR-146b-5p, hsa-miR-215-5p, and hsa-miR-485-5p. Validation in human samples revealed differential expression levels of hsa-miR-215-5p, hsa-miR-146b-5p, and hsa-miR-485-5p between the pneumoconiosis and control groups. Notably, in early-stage pneumoconiosis patients, the peripheral blood expression levels of hsa-miR-215-5p and hsa-miR-146b-5p were significantly upregulated, while hsa-miR-485-5p expression was significantly downregulated (Figure 6A).

Subsequently, logistic regression analysis was performed using log10-transformed expression values of hsa-miR-215-5p, hsa-miR-485-5p, and hsa-miR-146b-5p, with educational level, smoking, hypertension, and hyperlipidemia included as confounding factors. The results indicated that both hsa-miR-215-5p (OR = 1.966, 95% CI: 1.6938–2.2796, *p* < 0.001) and hsa-miR-146b-5p (OR = 1.9367, 95% CI: 1.697–2.201, *p* < 0.001) were risk factors for pneumoconiosis. This means that each log10-unit increase in their expression was associated with approximately 1.966-fold and 1.937-fold increases in disease risk, respectively. In contrast, hsa-miR-485-5p showed no statistically significant association (OR = 1.028, 95% CI: 0.9138–1.1258, *p* = 0.640), suggesting it is not a risk factor for pneumoconiosis (Table 2).

Receiver operating characteristic (ROC) curve analysis was performed to evaluate the diagnostic efficacy of hsa-miR-215-5p and hsa-miR-146b-5p for pneumoconiosis. The results demonstrated an AUC of 0.9563 (95% CI: 0.9261 to 0.9864) for hsa-miR-215-5p and of 0.8876 (95% CI: 0.8349 to 0.9404) for hsa-miR-146b-5p (*p* < 0.001 for both), indicating that both miRNAs possess a certain diagnostic value for pneumoconiosis.

## 4. Discussion

In this study, a mouse model of silicosis was employed to demonstrate that silica exposure leads to the progressive deterioration of lung function, induces the formation of characteristic fibrotic nodules, and drives profound transcriptomic remodeling within lung tissue. Compared to the control group, mice subjected to silica treatment exhibited significantly reduced respiratory frequency and Ti/Te ratio, indicating substantial lung function impairment. This finding is consistent with previous reports that particulate matter exposure reduces pulmonary ventilation capacity [38,39]. Such functional deficits are often attributable to physiological disturbances resulting from pulmonary inflammation and fibrosis [2,40]. Although tidal volume did not differ statistically between groups, values in the silica-treated mice remained lower than those in controls, suggesting possible restriction in lung compliance associated with structural injury or ongoing fibrotic remodeling [41]. These observations align with the classic pathophysiology of silicosis, in which fibrous scarring and nodule formation disrupt normal lung mechanics and ultimately compromise gas exchange.

Furthermore, whole-transcriptome sequencing identified extensive differentially expressed profiles, including 2950 mRNAs, 461 lncRNAs, 81 miRNAs, and 44 circRNAs. Notably, the number of upregulated genes significantly exceeded that of downregulated genes, strongly suggesting that the organism’s response to silica-induced injury is not merely suppressive or degenerative. Instead, it involves the active initiation of a complex program of gene activation and regulation to cope with persistent inflammatory and fibrotic stimulation. Further GO and KEGG enrichment analyses provided functional insights into these global changes. Differentially expressed genes were significantly enriched in classic pathways closely associated with pulmonary fibrosis [2,4,40], such as ECM–receptor interaction, PI3K-Akt signaling pathway, NF-κB signaling pathway, and TNF signaling pathway [42,43,44]. Numerous studies have indicated that, during the development of pulmonary fibrosis, inflammatory factors such as IL-1β, TNF-α, IL-6, and TGF-β1, together with related signaling pathways, including the inflammasome and NF-κB, promote fibroblast accumulation and proliferation and induce EMT, thereby collectively driving the abnormal deposition of ECM and contributing to the progression of fibrosis in silicosis [14,45]. GSEA further supplemented these findings by indicating the activation of pathways related to lysosomes, complement and coagulation cascades, and multiple pattern recognition receptors (Toll-like receptors, RIG-I-like receptors). In summary, silica dust triggers a coherent pathological cascade, progressing from early inflammatory recognition (via pattern recognition receptor pathways) to immune cell recruitment and activation (B cell receptor signaling, Th cell differentiation), followed by sustained inflammatory and profibrotic signal transduction (NF-κB, TNF, TGF-β signaling pathway), and ultimately leading to abnormal extracellular matrix deposition (ECM–receptor interaction) [4,14,46].

The competitive endogenous RNA (ceRNA) regulatory network plays a critical role in the development of silicosis [19,43]. For instance, during the complex transcriptomic remodeling triggered by silica exposure, lncRNAs and circRNAs can function as “molecular sponges” that competitively bind to miRNA response elements, thereby relieving miRNA-mediated repression of downstream target mRNAs. This interaction forms a dynamic and multi-dimensional “lncRNA/circRNA–miRNA–mRNA” regulatory axis that finely modulates the fibrotic process [35,47]. For example, circular RNA circPVT1 enhances silica exposure-induced epithelial–mesenchymal transition and migratory ability in pulmonary epithelial cells by acting as a molecular sponge for miR-497-5p, thereby upregulating the expression of the transcription factor TCF3 [29]. In silicosis-associated pulmonary fibrosis, upregulated LncRNA XIST promotes EMT in alveolar epithelial cells by directly sponging miR-101-3p, thereby upregulating the expression of the transcription factor ZEB1 [48]. In the NR8383/RLE-6TN co-culture system, LncRNA MRAK052509 promotes EMT by competitively sequestering miR-204-3p, thereby alleviating its inhibitory effect on the target gene TGF-βRI [49]. In a mouse model of pulmonary fibrosis and cellular experiments, lncRNA SNHG20 was found to drive fibroblast activation and pulmonary fibrotic progression by competitively sequestering miR-490-3p, thereby relieving its inhibitory effect on the target gene TGFBR1 [50]. During epithelial–fibroblast cross-talk, epithelial-derived IL-1α induces the expression of miR-146a-5p in fibroblasts, which in turn negatively regulates IL-8 production by targeting IRAK1 [51].

Thus, based on the identified differentially expressed molecules, we constructed a ceRNA regulatory network that provides critical mechanistic insights into how these molecules cooperate. For example, we found that circRNA (18_60522864_60572570) likely regulates the expression of the fibrotic core genes Fn1 and Fbln2 by sponging miR-468a-5p and miR-468b-5p. Additionally, we found that the ncRNA-mRNA regulatory networks in the ECM–receptor interaction, NF-κB, TNF, and PI3K-Akt signaling pathways all exhibited a multi-layered ceRNA regulatory pattern. For example, in the ECM–receptor interaction pathway, nine miRNAs, eight lncRNAs, and two circRNAs were identified to collectively regulate ten key genes. Similar regulatory structures were observed in the other three pathways. These findings indicate that, during silicosis progression, non-coding RNAs do not function in isolation but rather form an interconnected and functionally redundant regulatory network to cooperatively modulate the activation of fibrosis-related signaling pathways. Notably, this regulatory network displayed distinct “many-to-many” interactive characteristics: a single miRNA can target multiple genes, and a single gene can be coregulated by multiple non-coding RNAs. For instance, miR-486a-5p, miR-490-3p, and miR-1941-5p simultaneously regulate key genes such as Fn1, Spp1, and CCL3, while these miRNAs themselves are modulated by non-coding RNAs including circRNA_Cd4 and Gm42500. Furthermore, RT-qPCR validation supported the existence of this network. After SiO_2_ exposure, genes such as Fn1, Spp1, Nfkb2, and CCL3, along with non-coding RNAs including miR-146a-5p, Gm46224, and circRNA_18_60522864_60572570, were significantly upregulated, whereas miR-486a-5p, Neat1, and circRNA0010422 were significantly downregulated—consistent with the transcriptomic sequencing results. These expression changes experimentally confirm the cooperative regulation between non-coding RNAs and their target genes in key signaling pathways, providing important molecular evidence for elucidating how silica drives pulmonary fibrosis through epitranscriptional mechanisms. Concurrently, the protein–protein interaction (PPI) analysis of key mRNAs identified hub molecules such as *Gapdh*, *Fn1*, *Tlr2*, *Cd44*, *Spp1*, and *CCL3*, which occupy central positions in the network. These molecules play essential roles in core cellular processes including metabolism, matrix remodeling, and inflammatory initiation [4,40].

Currently, there are no effective therapeutic interventions for silicosis, and the disease remains nearly incurable following diagnosis. However, traditional diagnostic methods such as imaging and lung biopsy have limitations in identifying early-stage lesions, making the exploration of biomarkers for early diagnosis crucial. Studies indicate that miRNAs play a key regulatory role in the onset and progression of silicosis, and their significantly altered expression profiles make them highly promising biomarkers for diagnosis, prognosis, and treatment monitoring [30]. For instance, research has shown that miR-107, miR-125a-5p, miR-7219-3p, miR-23a-3p, miR-552-3p, and let-7i-5p may serve as biomarkers and therapeutic targets for the early diagnosis of silicosis, though clinical validation is still lacking. Additionally, in the peripheral blood leukocytes of silicosis patients, the expression of miR-19a is significantly downregulated. As a biomarker, it exhibits sensitivity and specificity as high as 95% in differential diagnosis, indicating its potential value for early screening [52]. In this study, based on miRNAs found to be significantly differentially expressed in animal models, we identified human homologous candidate molecules such as hsa-miR-146b-5p and hsa-miR-215-5p through stringent cross-species homology analysis. In an independent human cohort (62 pneumoconiosis patients, 73 controls), RT-qPCR validation results were highly consistent with the animal model data. In the peripheral blood of early-stage pneumoconiosis patients, the expression of hsa-miR-215-5p and hsa-miR-146b-5p was significantly upregulated. After further adjusting for potential confounding factors including education level, smoking, hypertension, and hyperlipidemia, multivariate logistic regression analysis revealed that hsa-miR-215-5p (OR = 1.966) and hsa-miR-146b-5p (OR = 1.9367) are independent risk factors for pneumoconiosis. Each logarithmic unit increase in their expression levels was associated with approximately a 1.97-fold and 1.94-fold increase in disease risk, respectively, demonstrating a clear dose–response relationship. This quantitative risk assessment is of significant value for identifying high-risk populations. ROC curve analysis also demonstrated excellent diagnostic potential. The AUC for hsa-miR-215-5p was as high as 0.956, indicating its strong ability to distinguish pneumoconiosis patients from healthy individuals; the AUC for hsa-miR-146b-5p was 0.888, also reflecting good diagnostic performance. These metrics surpass many traditional or investigational biomarkers [53,54,55]. Further analysis indicates that the predicted target genes of miR-215-5p, including NOX4, SLC39A6, BCL2L11, and CLIC5, are critically involved in cellular stress responses, programmed cell death, inflammation, and fibrotic processes [56,57,58]. For example, studies have shown that the compound trichlorobenzylamine directly binds to the key residues of NOX4 (PHE354/THR355), inhibiting its activity. This leads to the downregulation of the ARG1/OAT pathway and the suppression of TGF-β/SMADs signaling, while simultaneously enhancing the Nrf2-Keap1 antioxidant system [56]. Consequently, the metabolic dysregulation of arachidonic acid, arginine, and proline is restored, mitigating oxidative stress and reducing collagen deposition. Similarly, KCTD15, a potential target of miR-146b-5p, participates in the regulation of multiple signaling pathways such as NF-κB and has been linked to several disease states [59,60]. Experimental findings demonstrate that miR-146b-5p directly suppresses HMGB1 expression and nuclear translocation, thereby inhibiting downstream inflammatory mediator production [60]. In summary, these differentially expressed miRNAs may serve not only as biomarkers of disease progression but may also uncover molecular pathways that represent promising therapeutic targets. Further functional studies will be necessary to validate these observations and clarify their translational relevance.

## 5. Conclusions

In this study, following a systematic research approach of “phenomenon observation–mechanism exploration–clinical validation” has progressively highlighted the crucial role of non-coding RNAs in the development of silicosis. The constructed ceRNA regulatory network offers a novel perspective and valuable insights into the molecular and pathological mechanisms of silicosis. Additionally, the identified circulating miRNA biomarkers (hsa-miR-215-5p and hsa-miR-146b-5p) provide a potentially translatable strategy for addressing the clinical challenge of early pneumoconiosis diagnosis.

## Figures and Tables

**Table 1:** Baseline characteristics of pneumoconiosis and control groups.

Characteristics	Pneumoconiosis (*n* = 62)	Control (*n* = 73)	t/χ^2^	*p*-Value
Age, year, mean ± SD	50.69 ± 3.43	49.86 ± 3.20	−1.454	0.148
Waistline, cm, mean ± SD	88.59 ± 10.43	90.34 ± 7.68	−1.092	0.277
Hipline, cm, mean ± SD	98.70 ± 6.53	99.08 ± 6.12	−0.348	0.728
BMI, kg/m^2^, mean ± SD	24.17 ± 4.13	25.35 ± 3.64	−1.737	0.085
Education level			43.317	<0.001
College degree and above	17 (27.42%)	61 (83.56%)		
High school and below	45 (72.58%)	12 (16.44%)		
Shift work			8.11	0.004
Yes	47 (75.8%)	38 (52.1%)		
No	15 (24.2%)	35 (47.9%)		
Annual household income			0.202	0.653
<CNY 200,000	27 (43.54%)	29 (39.73%)		
≥CNY 200,000	35 (56.45%)	44 (60.27%)		
Hypertension			5.454	0.020
Yes	22 (35.5%)	13 (17.8%)		
No	40 (64.5%)	60 (82.2%)		
Hyperlipidemia			7.992	0.005
Yes	15 (24.2%)	5 (6.8%)		
No	47 (75.8%)	68 (93.2%)		
Diabetes			0.791	0.374
Yes	8 (12.90%)	6 (8.22%)		
No	54 (87.10%)	67 (91.78%)		
Smoking			3.638	0.056
Yes	52 (83.87%)	51 (69.86%)		
No	10 (16.13%)	22 (30.14%)		
Drinking			3.479	0.062
Yes	24 (38.71%)	40 (54.80%)		
No	38 (61.29%)	33 (45.20%)		
FEV1%	70.90 ± 16.98	81.12 ± 19.74	3.194	0.002
FEV1/FVC%	88.06 ± 11.43	91.45 ± 11.75	1.688	0.094
FVC%	81.36 ± 19.39	89.33 ± 20.20	2.328	0.021

**Table 2:** Associations of miRNAs with pneumoconiosis risk.

ID	OR (95%CI)	*p*
hsa-miR-215-5p	1.966 (1.6938,2.2796)	<0.001
hsa-miR-485-5p	1.028 (0.9138,1.2585)	0.640
hsa-miR-146b-5p	1.9367 (1.697–2.201)	<0.001

The confounding factors include education level, shift work, hypertension, hyperlipidemia, and smoking.

## Data Availability

The original data presented in the study are openly available in the NCB at accession number PRJNA1376149.

## Supplementary Materials

The following supporting information can be downloaded at: https://www.mdpi.com/article/10.3390/toxics14010063/s1, Figure S1: Gene Set Enrichment Analysis (GSEA) of differentially expressed genes. The analysis was performed based on RNA sequencing data. NES, Normalized Enrichment Score. Figure S2: The ceRNA networks were constructed for differentially expressed miRNAs, mRNAs, lncRNAs, and circRNAs. Figure S3: The PPI network for 295 key mRNA genes. Table S1: The information of the study population. Table S2: Primers of quantitative real-time PCR (qRT-PCR). Table S3: Sequencing data for longRNA sequencing (mRNA, lncRNA, circRNA) and mapping rate. Table S4: Sequencing data for smallRNA and mapping rate. Table S5: Differentially expressed genes (DEmRNAs) (NS = CK, MS = SiO_2_). Table S6: Differentially expressed miRNAs (NS = CK, MS = SiO_2_). Table S7: Differentially expressed lncRNAs (NS = CK, MS = SiO_2_). Table S8: Differentially expressed circRNAs (NS = CK, MS = SiO_2_). Table S9: lncRNA-miRNA-mRNA correspondence networks. Table S10: circRNA-miRNA-mRNA correspondence networks. Table S11: The information of 295 key mRNA genes.

## Abbreviations

The following abbreviations are used in this manuscript:

ceRNA	competing endogenous RNA
EMT	epithelial–mesenchymal transition
CK	control group
SiO_2_	silica-treated group
Ti	inspiratory time
Tv	tidal volume
Te	expiratory time
FVC	forced vital capacity
FEV1	forced expiratory volume in one second
FVC%	percentage of predicted forced vital capacity
FEV1%	percentage of predicted forced expiratory volume in one second
H&E	hematoxylin and eosin
RT-qPCR	quantitative real-rime PCR
ROC	receiver operating characteristic
DEmRNAs	differentially expressed genes
AUC	area under the curve
PI3K	phosphatidylinositol 3-kinase
NF-κB	nuclear factor kappa-light-chain-enhancer of activated B cell
mTOR	mechanistic target of rapamycin
PTEN	phosphatase and yensin homolog deleted on chromosome ten
BMI	body mass index
EggNOG	evolutionary genealogy of genes: non-supervised orthologous groups
GO	gene ontology
KEGG	kyoto encyclopedia of genes and genomes

## References

[1] Howlett P., Gan J., Lesosky M., Feary J. (2024). Relationship between cumulative silica exposure and silicosis: A systematic review and dose-response meta-analysis. Thorax.

[2] Leung C.C., Yu I.T.S., Chen W. (2012). Silicosis. Lancet.

[3] Vanka K.S., Shukla S., Gomez H.M., James C., Palanisami T., Williams K., Chambers D.C., Britton W.J., Ilic D., Hansbro P.M. (2022). Understanding the pathogenesis of occupational coal and silica dust-associated lung disease. Eur. Respir. Rev..

[4] Yang B., Liu X., Peng C., Meng X., Jia Q. (2025). Silicosis: From pathogenesis to therapeutics. Front. Pharmacol..

[5] Hoy R.F., Jeebhay M.F., Cavalin C., Chen W., Cohen R.A., Fireman E., Go L.H.T., León-Jiménez A., Menéndez-Navarro A., Ribeiro M. (2022). Current global perspectives on silicosis—Convergence of old and newly emergent hazards. Respirology.

[6] Huang X., Liang R., Liu Y., Yu L., Yang M., Shang B., Zhang H., Ma J., Chen W., Wang D. (2024). Incidence, mortality, and disability-adjusted life years due to silicosis worldwide, 1990–2019: Evidence from the global burden of disease study 2019. Environ. Sci. Pollut. Res..

[7] Liu X., Jiang Q., Wu P., Han L., Zhou P. (2023). Global incidence, prevalence and disease burden of silicosis: 30 years’ overview and forecasted trends. BMC Public Health.

[8] Wang H., Ye Q., Zhang H., Sun X., Li T. (2023). Prevention and treatment of pneumoconiosis in the context of healthy China 2030. China CDC Wkly.

[9] Rupani M.P. (2023). Challenges and opportunities for silicosis prevention and control: Need for a national health program on silicosis in India. J. Occup. Med. Toxicol..

[10] Bell J.L., Mazurek J.M. (2020). Trends in Pneumoconiosis Deaths—United States, 1999–2018. Morb. Mortal. Wkly. Rep..

[11] Li T., Yang X., Xu H., Liu H., Moitra S. (2022). Early Identification, Accurate Diagnosis, and Treatment of Silicosis. Can. Respir. J..

[12] Li S., Zhao J., Han G., Zhang X., Li N., Zhang Z. (2023). Silicon dioxide-induced endoplasmic reticulum stress of alveolar macrophages and its role on the formation of silicosis fibrosis: A review article. Toxicol. Res..

[13] Liu T.-T., Sun H.-F., Han Y.-X., Zhan Y., Jiang J.-D. (2024). The role of inflammation in silicosis. Front. Pharmacol..

[14] Adamcakova J., Mokra D. (2021). New Insights into Pathomechanisms and Treatment Possibilities for Lung Silicosis. Int. J. Mol. Sci..

[15] Kawasaki H. (2015). A mechanistic review of silica-induced inhalation toxicity. Inhal. Toxicol..

[16] Ma Y., Liang Q., Wang F., Yan K., Sun M., Lin L., Li T., Duan J., Sun Z. (2022). Silica nanoparticles induce pulmonary autophagy dysfunction and epithelial-to-mesenchymal transition via p62/NF-κB signaling pathway. Ecotoxicol. Environ. Saf..

[17] DU Y., Huang F., Guan L., Zeng M. (2023). Role of PI3K/Akt/mTOR pathway-mediated macrophage autophagy in affecting the phenotype transformation of lung fibroblasts induced by silica dust exposure. J. Cent. South Univ. Med. Sci..

[18] Tao H., Zhao H., Ge D., Liao J., Shao L., Mo A., Hu L., Xu K., Wu J., Mu M. (2022). Necroptosis in pulmonary macrophages promotes silica-induced inflammation and interstitial fibrosis in mice. Toxicol. Lett..

[19] Yin H., Xie Y., Gu P., Li W., Zhang Y., Yao Y., Chen W., Ma J. (2022). The emerging role of epigenetic regulation in the progression of silicosis. Clin. Epigenetics.

[20] Li Y., Cheng Z., Fan H., Hao C., Yao W. (2022). Epigenetic changes and functions in pneumoconiosis. Oxidative Med. Cell. Longev..

[21] Zhang X., Jia X., Mei L., Zheng M., Yu C., Ye M. (2016). Global DNA methylation and PTEN hypermethylation alterations in lung tissues from human silicosis. J. Thorac. Dis..

[22] Li J., Yao W., Zhang L., Bao L., Chen H., Wang D., Yue Z., Li Y., Zhang M., Hao C. (2017). Genome-wide DNA methylation analysis in lung fibroblasts co-cultured with silica-exposed alveolar macrophages. Respir. Res..

[23] Ferrer J., Dimitrova N. (2024). Transcription regulation by long non-coding RNAs: Mechanisms and disease relevance. Nat. Rev. Mol. Cell Biol..

[24] Nemeth K., Bayraktar R., Ferracin M., Calin G.A. (2024). Non-coding RNAs in disease: From mechanisms to therapeutics. Nat. Rev. Genet..

[25] Li C., Ni Y.-Q., Xu H., Xiang Q.-Y., Zhao Y., Zhan J.-K., He J.-Y., Li S., Liu Y.-S. (2021). Roles and mechanisms of exosomal non-coding RNAs in human health and diseases. Signal Transduct. Target. Ther..

[26] Mattick J.S., Amaral P.P., Carninci P., Carpenter S., Chang H.Y., Chen L.-L., Chen R., Dean C., Dinger M.E., Fitzgerald K.A. (2023). Long non-coding RNAs: Definitions, functions, challenges and recommendations. Nat. Rev. Mol. Cell Biol..

[27] Du M., Wang J., Li W., Mu M., Zhu F., Ye D. (2023). Research and progress of miRNA as potential biomarkers for pneumoconiosis. Chin. J. Dis. Control. Prev..

[28] Zhang B., Wang E., Zhou S., Han R., Wu W., Sun G., Cao C., Wang R. (2024). RELA-mediated upregulation of LINC03047 promotes ferroptosis in silica-induced pulmonary fibrosis via SLC39A14. Free Radic. Biol. Med..

[29] Zhou S., Li Y., Sun W., Ma D., Liu Y., Cheng D., Li G., Ni C. (2024). circPVT1 promotes silica-induced epithelial-mesenchymal transition by modulating the miR-497-5p/TCF3 axis. J. Biomed. Res..

[30] Gupta G., Goyal A., Ilma B., Rekha M.M., Nayak P.P., Kaur M., Khachi A., Goyal K., Rana M., Rekha A. (2025). Exosomal miRNAs as biomarkers and therapeutic targets in silicosis-related lung fibrosis. Mol. Biol. Rep..

[31] Gao X., Xu D., Li S., Wei Z., Li S., Cai W., Mao N., Jin F., Li Y., Yi X. (2020). Pulmonary silicosis alters microRNA expression in rat lung and miR-411-3p exerts anti-fibrotic effects by inhibiting MRTF-A/SRF signaling. Mol. Ther. Nucleic Acids.

[32] Pang X., Shi H., Chen X., Li C., Shi B., Yeo A.J., Lavin M.F., Jia Q., Shao H., Zhang J. (2022). miRNA-34c-5p targets Fra-1 to inhibit pulmonary fibrosis induced by silica through p53 and PTEN/PI3K/Akt signaling pathway. Environ. Toxicol..

[33] Yang G., Tian Y., Li C., Xia J., Qi Y., Yao W., Hao C. (2022). LncRNA UCA1 regulates silicosis-related lung epithelial cell-to-mesenchymal transition through competitive adsorption of miR-204-5p. Toxicol. Appl. Pharmacol..

[34] Surendran A., Huang C., Liu L. (2024). Circular RNAs and their roles in idiopathic pulmonary fibrosis. Respir. Res..

[35] Liu T., Su X., Kong X., Dong H., Wei Y., Wang Y., Wang C. (2024). Whole transcriptome sequencing identifies key lncRNAs, circRNAs, and mRNAs for exploring the pathogenesis and therapeutic target of mouse pneumoconiosis. Gene.

[36] Li B., Mu M., Sun Q., Cao H., Liu Q., Liu J., Zhang J., Xu K., Hu D., Tao X. (2021). A suitable silicosis mouse model was constructed by repeated inhalation of silica dust via nose. Toxicol. Lett..

[37] (2014). Standard for Identify Work Ability—Gradation of Disability Caused by Work-Related Injuries and Occupatiaonal Diseases.

[38] Janssen L.M.F., Lemaire F., Marain N.F., Ronsmans S., Heylen N., Vanstapel A., Velde G.V., Vanoirbeek J.A.J., Pollard K.M., Ghosh M. (2024). Differential pulmonary toxicity and autoantibody formation in genetically distinct mouse strains following combined exposure to silica and diesel exhaust particles. Part. Fibre Toxicol..

[39] Gamble J.F., Hessel P.A., Nicolich M. (2004). Relationship between silicosis and lung function. Scand. J. Work Environ. Health.

[40] Weissman D.N., Tallaksen R.J. (2022). Silicosis. Modern Occupational Diseases: Diagnosis, Epidemiology, Management and Prevention.

[41] Hallett S., Toro F., Ashurst J.V. (2018). Physiology, Tidal Volume.

[42] Jing Z., Zhuoya J., Juan W., Lu L. (2022). The role of TLR4/NF-κB signaling pathway in the regulation of lipid metabolism of alveolar macrophages in a model of silicosis induced by SiO_2_. Chin. J. Clin. Anat..

[43] Choi J.-K., Lee S.-G., Lee J.Y., Nam H.-Y., Lee W.-k., Lee K.-H., Kim H.J., Lim Y. (2005). Silica Induces Human Cyclooxygenase-2 Gene Expression Through the NF-kB Signaling Pathway. J. Environ. Pathol. Toxicol. Oncol..

[44] Qian Q.-Z., Cao X.-K., Liu H.-Y., Zheng G.-Y., Qian Q.-Q., Shen F.-H. (2017). TNFR/TNF-α signaling pathway regulates apoptosis of alveolar macrophages in coal workers’ pneumoconiosis. Oncotarget.

[45] Komai M., Mihira K., Shimada A., Miyamoto I., Ogihara K., Naya Y., Morita T., Inoue K., Takano H. (2019). Pathological Study on Epithelial-Mesenchymal Transition in Silicotic Lung Lesions in Rat. Vet. Sci..

[46] Naim M.J. (2025). Pulmonary Fibrosis: Causes, Development, Diagnosis, and Treatment with Emphasis on Murine and in vitro Models. Curr. Respir. Med. Rev..

[47] Ma J., Han B., Yang Y., Zhang Y., Cao M., Cao W., Zhang W., Cheng M., Cui G., Du Z. (2025). CircRNA-mediated ceRNA network: Micron-sized quartz silica particles induce apoptosis in primary human airway epithelial cells. Toxicol. Mech. Methods.

[48] Li C., Xia J., Yao W., Yang G., Tian Y., Qi Y., Hao C. (2022). Mechanism of LncRNA XIST/ miR-101–3p/ZEB1 axis in EMT associated with silicosis. Toxicol. Lett..

[49] Xuan L., Zi-ming J., Xue-yan T., Wen-xuan H., Fa-xuan W. (2024). LncRNA MRAK052509 competitively adsorbs miR-204-3p to regulate silica dust-induced EMT process. Environ. Toxicol..

[50] Cheng D., Xu Q., Liu Y., Li G., Sun W., Ma D., Ni C. (2021). Long noncoding RNA-SNHG20 promotes silica-induced pulmonary fibrosis by miR-490-3p/TGFBR1 axis. Toxicology.

[51] Osei E.T., Florez-Sampedro L., Tasena H., Faiz A., Noordhoek J.A., Timens W., Postma D.S., Hackett T.L., Heijink I.H., Brandsma C.-A. (2017). miR-146a-5p plays an essential role in the aberrant epithelial–fibroblast cross-talk in COPD. Eur. Respir. J..

[52] Yang Z., Li Q., Yao S., Zhang G., Xue R., Li G., Wang Y., Wang S., Wu R., Gao H. (2016). Down-Regulation of miR-19a as a Biomarker for Early Detection of Silicosis. Anat. Rec..

[53] Lombardi E.M.S., Mizutani R.F., Terra-Filho M., Ubiratan de Paula S. (2023). Biomarkers related to silicosis and pulmonary function in individuals exposed to silica. Am. J. Ind. Med..

[54] Wang Z., Zhang J., Wang T., Liu Z., Zhang W., Sun Y., Wu X., Shao H., Du Z. (2024). The value of single biomarkers in the diagnosis of silicosis: A meta-analysis. iScience.

[55] Nong Q., Zhu X., Zhong L., Li Y., Yao L., Hu Z., Wu S., Zou Z., Li C., Liu Z. (2025). Serum Krebs von den Lungen-6 as a potential biomarker for early diagnosis of silicosis: A case-control study. BMC Pulm. Med..

[56] Liu F., Yao Y., Guo C., Dai P., Huang J., Peng P., Wang M., Dawa Z., Zhu C., Lin C. (2024). Trichodelphinine A alleviates pulmonary fibrosis by inhibiting collagen synthesis via NOX4-ARG1/TGF-β signaling pathway. Phytomedicine.

[57] Boina B.B., Jing Z., Harpreet S., Yutong Z. (2025). Intracellular Chloride Channels: A Rising Target in Lung Disease Research. J. Respir. Biol. Transl. Med..

[58] Fu T., Tian H., Rong H., Ai P., Li X. (2023). LncRNA PVT1 induces apoptosis and inflammatory response of bronchial epithelial cells by regulating miR-30b-5p/BCL2L11 axis in COPD. Genes Environ..

[59] Smaldone G., Pecoraro G., Pane K., Franzese M., Ruggiero A., Vitagliano L., Salvatore M. (2023). The Oncosuppressive Properties of KCTD1: Its Role in Cell Growth and Mobility. Biology.

[60] Cheng J., Tian X., Wu C., Wang J., Liu H., Cheng S., Sun H. (2025). MiR-146b-5p inhibits Candida albicans-induced inflammatory response through targeting HMGB1 in mouse primary peritoneal macrophages. Heliyon.

