# Supplementary material for: Integrated ceRNA Network Analysis in Silica-Induced Pulmonary Fibrosis and Discovery of miRNA Biomarkers
Source: Toxics. 2026 Jan 9;14(1):63. doi: 10.3390/toxics14010063 (PMC12845774; doi:10.3390/toxics14010063)
Supplement: Supplementary file 1 [file toxics-14-00063-s001.zip › Figures S1-S3.pdf]

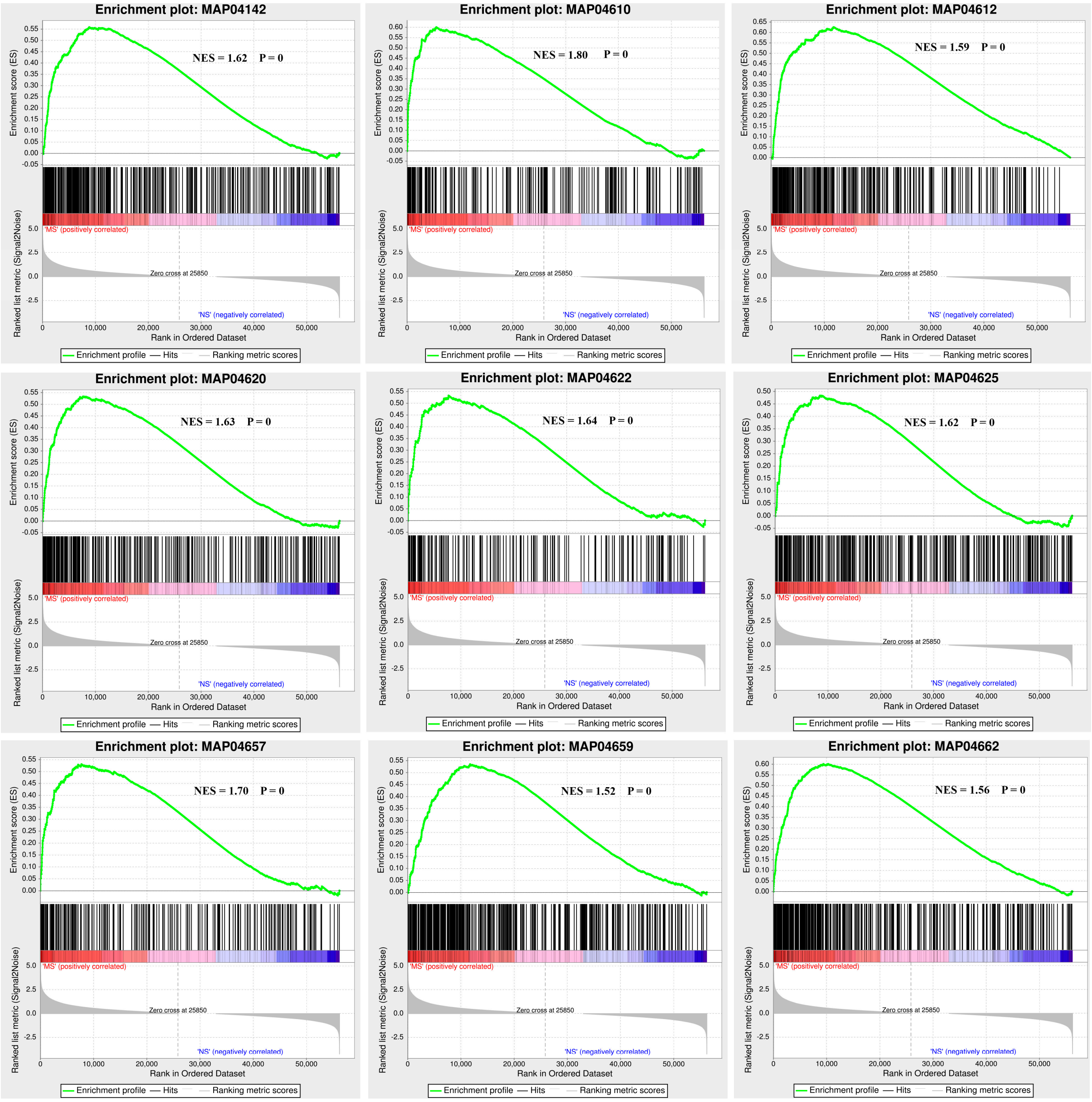

Figure S1. Gene Set Enrichment Analysis (GSEA) of Differentially Expressed Genes. The analysis was performed based on RNA sequencing data. NES, Normalized Enrichment Score.

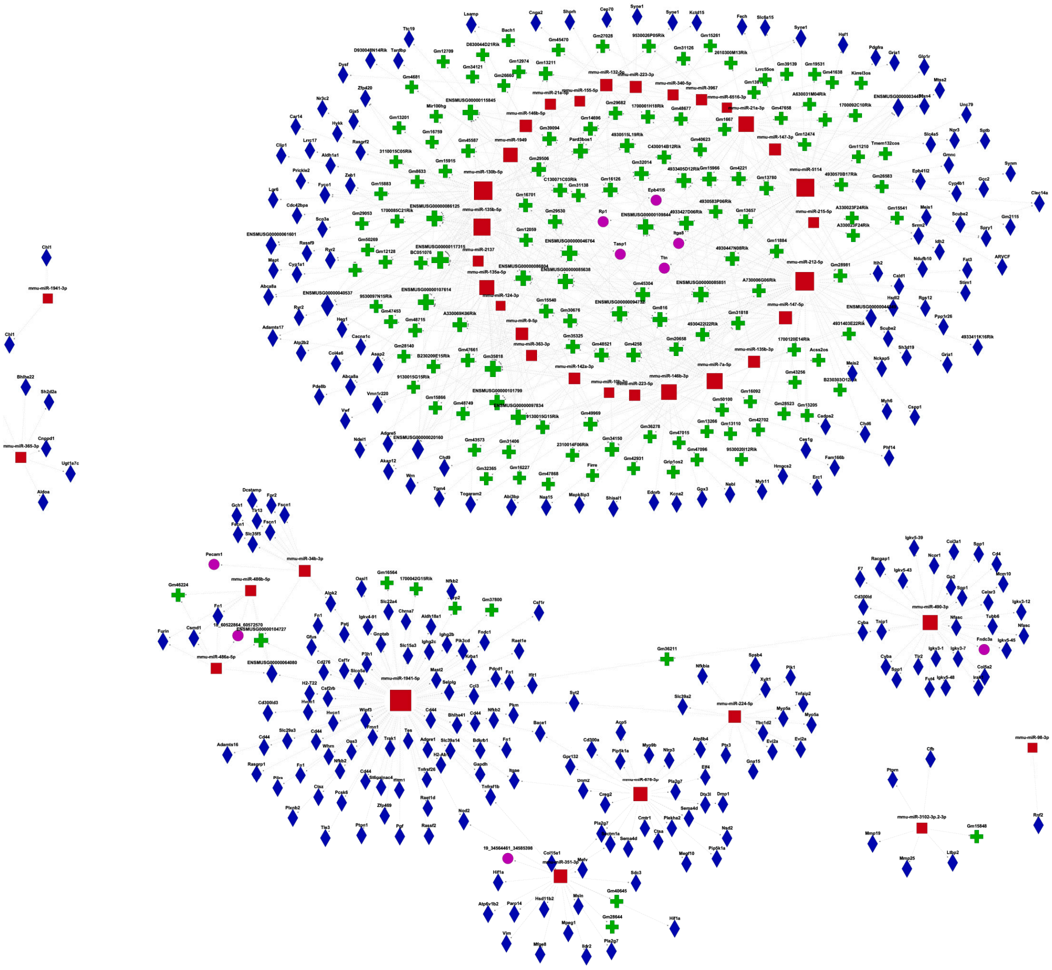

Figure S2. The ceRNA networks was constructed of differentially expressed miRNAs, mRNAs, lncRNAs, and circRNAs. Red squares represent miRNAs, green crosses represent lncRNAs, purple circles represent circRNAs, and blue diamonds represent mRNAs.

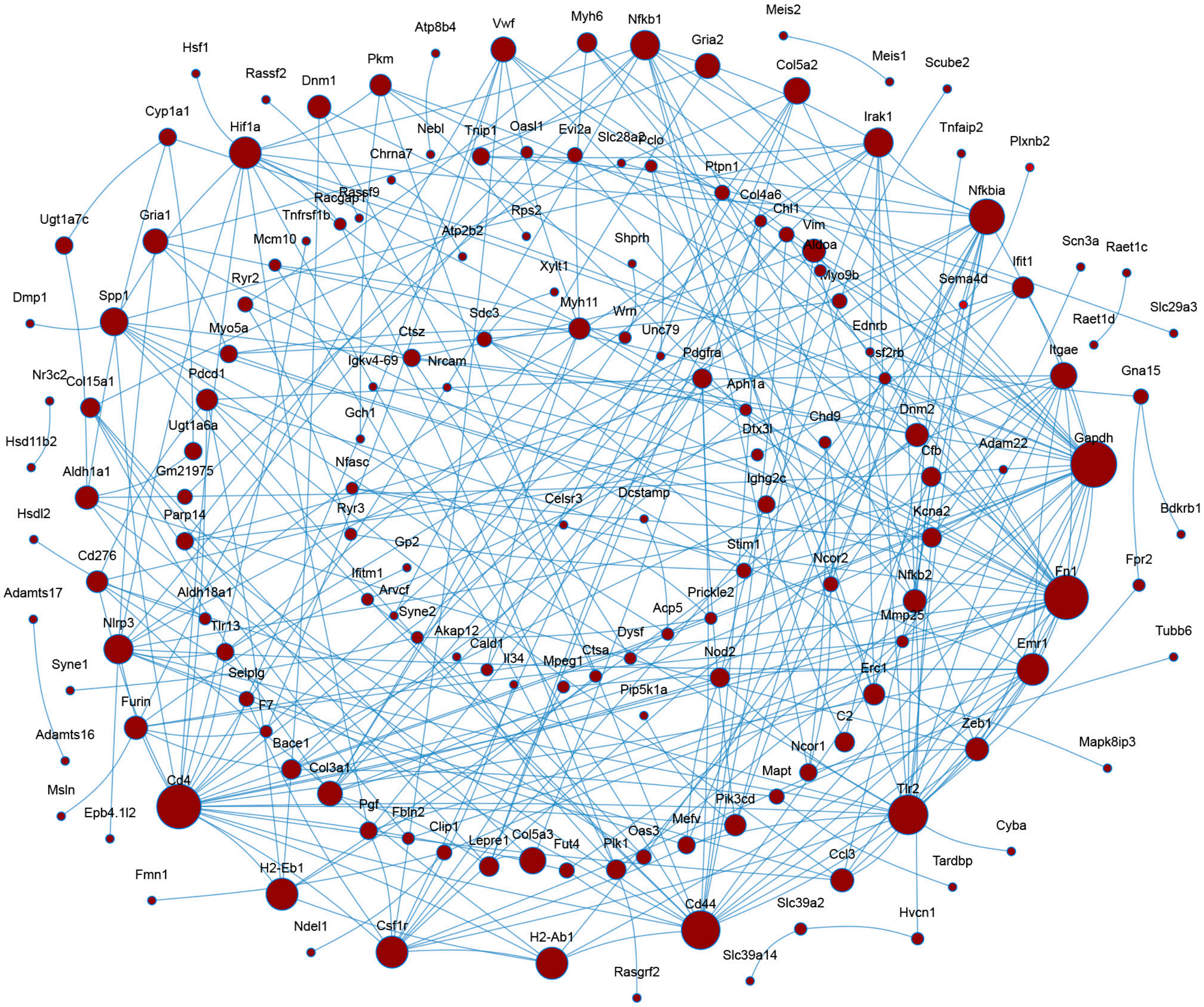

Figure S3. The PPI network for 295 key mRNA genes.
